# Depressive symptomatology among residents of the rural area of a city in Southern Brazil

**DOI:** 10.11606/S1518-8787.2018052000266

**Published:** 2018-09-13

**Authors:** Roberta Hirschmann, Ana Paula Gomes, Helen Gonçalves

**Affiliations:** I Universidade Federal de Pelotas . Faculdade de Medicina . Programa de Pós-Graduação em Epidemiologia . Pelotas , RS , Brasil; II Universidade Federal de Pelotas . Faculdade de Medicina . Departamento de Medicina Social . Pelotas , RS , Brasil

**Keywords:** Adult, Depressive Disorder, epidemiology, Risk Factors, Socioeconomic Factors, Rural Population, Adulto, Transtorno Depressivo, epidemiologia, Fatores de Risco, Fatores Socioeconômicos, População Rural

## Abstract

**OBJECTIVE:**

To evaluate the prevalence and demographic, socioeconomic, behavioral and health factors associated with depressive symptomatology in rural residents.

**METHODS:**

This is a population-based, cross-sectional study with a representative sample of 1,453 residents aged 18 years or over of the rural area of the city of Pelotas, State of Rio Grande do Sul, Brazil. We used the Edinburgh Postnatal Depression Scale to evaluate depressive symptomatology, considering the cutoff point ≥ 8 points. We evaluated the association between the outcome and the independent variables using Poisson regression.

**RESULTS:**

The prevalence of depressive symptomatology was 35.4% (95%CI 31.5–39.3). After adjustment, the depressive symptomatology was higher among women (PR *=* 1.77, 95%CI 1.46–2.15), individuals with low education level (0–4 years of study) (PR *=* 1.62, 95%CI 1.22–2.16), worse socioeconomic conditions (classes D or E) (PR *=* 1.49, 95%CI 1.22–1.83), and with chronic diseases (PR *=* 1.74, 95%CI 1.24–2.45).

**CONCLUSIONS:**

The high prevalence of depressive symptomatology in rural residents indicates the relevance of depression as an important public health problem in this population. Specific attention should be aimed at the subgroups that presented the highest prevalence of symptomatology.

## RESUMO

**OBJETIVO:**

Avaliar a prevalência e os fatores demográficos, socioeconômicos, comportamentais e de saúde associados à sintomatologia depressiva em moradores na zona rural.

**MÉTODOS:**

Estudo transversal de base populacional realizado com amostra representativa de 1.453 residentes na zona rural do município de Pelotas, RS, com 18 anos ou mais. Utilizou-se a Escala de Depressão Pós-Natal de Edimburgo para avaliar a sintomatologia depressiva, considerando ponto de corte ≥ 8 pontos. A associação entre o desfecho e as variáveis independentes foi avaliada por regressão de Poisson.

**RESULTADOS:**

A prevalência de sintomatologia depressiva foi de 35,4% (IC95% 31,5–39,3). Após o ajuste, a sintomatologia depressiva foi maior entre as mulheres (RP = 1,77; IC95% 1,46–2,15), indivíduos com baixa escolaridade (0–4 anos completos de estudo) (RP = 1,62; IC95% 1,22–2,16), pior condição socioeconômica (classes D ou E) (RP = 1,49; IC95% 1,22–1,83) e com doenças crônicas (RP = 1,74; IC95% 1,24–2,45).

**CONCLUSÕES:**

A alta prevalência de sintomatologia depressiva em moradores rurais indica a relevância da depressão como importante problema de saúde pública também nessa população. Atenção específica deve ser direcionada aos subgrupos que apresentaram as maiores prevalências de sintomatologia.

## INTRODUCTION

Non-communicable chronic diseases, including mental disorders, are one of the major health problems in the world given the high rates of morbidity and mortality ^1^ . Among mental disorders, the major depressive disorder affects a large part of the world population and it is one of the main causes of years lived with disability ^2^ .

Depression, a multifactorial disease, is diagnosed in the presence of symptoms for at least two weeks, such as: depressed mood most of the time, loss of pleasure in routine activities, excessive guilt, difficulty concentrating, loss of energy, sleep disorders, changes in weight in the absence of a new diet, and recurrent ideas of death or suicide ^3^ . If untreated, it tends to assume a chronic nature causing numerous damages to the individual and to the persons around them ^2^ .

In a recent meta-analysis, including 27 studies with Brazilian adults living predominantly in urban areas, the prevalence of depressive symptoms (evaluated by screening instruments or self-report) and major depressive disorder in the previous year was 14% and 8%, respectively ^4^ . Knowledge is quite limited as to the prevalence of this disease in the rural population since most studies evaluate the urban population. The 2013 Brazilian National Health Survey (PNS) ^5^ also evaluated some of the Brazilian rural population and it identified that 5.6% of the rural residents reported having received a diagnosis of depression by a physician or mental health professional, while 3.4% had a probable diagnosis of the disease. The highest percentages of depression recorded in rural areas in this study were in the Southeast (4.1%) and South (4.0%) regions ^6^ . Studies carried out in rural areas of high-income countries, using an instrument to evaluate depressive symptomatology in the previous week, have found distinct prevalences, between 9.3% and 31%, and this value is higher among women, individuals without partners, and among those with lower education level ^7^ ^,^ ^8^ .

The reduced number of studies carried out in rural areas hinders the access to information on this and other diseases and health interventions. This study aimed to estimate the prevalence and factors associated with depressive symptomatology in individuals living in rural areas.

## METHODS

This is a cross-sectional, population-based study carried out between January and June 2016, with a representative sample of individuals aged 18 years or over living in the rural area of the city of Pelotas, State of Rio Grande do Sul, Brazil. The study is part of a research consortium, led by master’s students of the Graduate Program in Epidemiology of Universidade Federal de Pelotas, whose methodology has been described by Barros et al. ^9^ The consortium has investigated subjects related to the health of the rural population, which are better detailed in the methodological article of this supplement ^10^ .

The eight districts in the rural area of Pelotas were part of the study. Twenty-four of the 50 rural census tracts were drawn according to the ratio number of permanent households per district; the final sample consisted of 30 households per tract. The data collection instrument included questions about demographic, socioeconomic and occupational, behavioral, and health characteristics. All residents in the households sampled and in the defined age group were invited to participate. For this study, we considered the following exclusion criteria: individuals with cognitive or mental disabilities, hospitalized, or institutionalized during the collection period, those who did not speak or did not understand Portuguese, and those who could not answer the depression questionnaire by themselves. We defined as refusals the individuals who did not agree to participate and as losses the individuals who were not found after at least three attempts of personal contact, at different days and times.

To calculate the sample size for this study, we considered a prevalence of 20% for depressive symptomatology, a 95% confidence interval, a margin of error of three percentage points, a delineation effect of 1.5, and an increase of 10% for losses and refusals, which amounted to 1,127 individuals as the minimum sample. For the study of associations, we later performed power calculations because of the unavailability of the necessary parameters to calculate the sample size beforehand.

During fieldwork, the database was checked weekly to identify possible inconsistencies and to ensure the overall quality of the data. For logistical reasons, quality control was carried out by phone using the reapplication of a reduced version of the questionnaire, with 10 questions, in 10% of the sample randomly selected. To calculate the agreement, the question of the instrument used to evaluate depressive symptomatology could not be used as it is a temporal question; in this case, we used the question “Do you know how to read and write?” and we obtained a Kappa coefficient of 0.76.

Depressive symptomatology was evaluated using the Edinburgh Postnatal Depression Scale (EPDS) ^11^ , which has been validated for the general adult population by Matijasevich et al. ^12^ The EPDS has 10 questions with four answer options, with a score between zero and three and a maximum of 30 points. According to the validation study ^12^ , the cutoff point of eight or more points is the most adequate for the screening of depressive symptomatology, with a sensitivity of 80% (95%CI 64.4–90.9), specificity of 87% (95%CI 83.3–90.1), positive predictive value of 37.6% (95%CI 27.4–48.8), and accuracy of 83.5% (95%CI 77.0–90.0). This cutoff point (≥ 8 points) indicates the presence of important factors of depressive symptomatology. We highlight that the positive predictive value was 57.6% for the cutoff point ≥ 13 points, proposed by Matijasevich et al. ^12^ , which indicates an increase of 20 percentage points (pp) – when compared to the cutoff point ≥ 8 – on the ability to detect correctly individuals with major a depressive episode (MDE) among those who presented positive EPDS. Therefore, we performed additional analyses also assuming this cutoff point, and the outcome was denominated as “probable MDE”, as the clinical interview is the gold standard.

The independent variables used in this study were: age (18–29; 30–39; 40–49; 50–59; ≥ 60 years); sex (male; female); race [white; black; brown; yellow; indigenous (recategorized in two groups: white; black, brown, or other)]; marital status (married or living with a partner; divorced, separated, or widowed; single); education level in complete years (0–4; 5–8; ≥ 9); socioeconomic class, collected according to the Brazilian Association of Research Companies (ABEP) ^13^ and categorized as A or B, C, and D or E; workplace (urban area; rural area; rural and urban area); rural worker (yes; no), being considered as rural worker the individual who carried out some daily or frequent work related to agriculture, livestock, and fishing in the rural area; time living in the rural area (50% of life *=* short time; 50%–99% of life *=* long time; 100% of life *=* always); number of residents in the household (1; 2; 3; 4; ≥ 5); leisure physical activity in minutes/week, evaluated by the Global Physical Activity Questionnaire (GPAQ) ^14^ and categorized as active (≥ 150 minutes) and insufficiently active ( *<* 150 minutes); smoking (yes; no), being considered as smokers those who smoked one or more cigarettes per day for at least one month or those who reported having quit smoking for less than a month; and number of morbidities, from reports of medical diagnosis of hypertension, diabetes, and heart disease (0; 1; 2; 3). The pattern of alcohol consumption, assessed by the Alcohol Use Disorder Identification Test (AUDIT) ^15^ , was categorized as low-risk (0–7 points) and at-risk (≥ 8 points) drinking.

We analyzed the data using the software Stata, version 14.0. We performed crude and adjusted analyses to examine the association between depressive symptomatology and exposure variables using Poisson regression, with robust adjustment for variance, and we obtained the prevalence ratios (PR) and 95% confidence intervals (95%CI).

We performed the adjusted analysis according to the hierarchical model, which was developed in three levels as shown in the Figure . In order to obtain the estimates and the p-value of the variable of socioeconomic class, we removed the variable of education level from the model, and we did not use it for adjustment as it presented collinearity. In the statistical modeling, we used the backward selection strategy, in which we kept the variables with p *<* 0.20 in the model to control for confounding factors, and we adopted a level of significance of 5%. The interaction test did not show evidence of effect modification according to sex; therefore, we present the combined estimates. The complex sampling process was considered in all analyses using the *svy* command.

**Figure:**
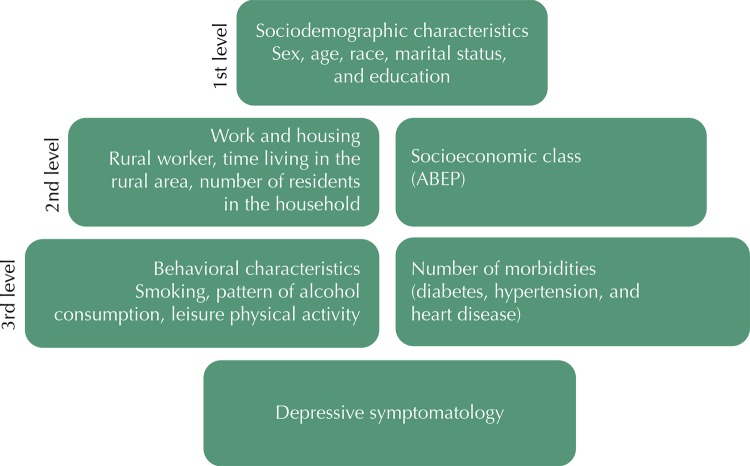
Model of hierarchical analysis for depressive symptomatology among rural residents. Pelotas, State of Rio Grande do Sul, Brazil, 2016.

ABEP: *Associação Brasileira de Empresas de Pesquisa* (Brazilian Association of Research Companies)

The study was approved by the Research Ethics Committee of the Faculdade de Medicina of the Universidade Federal de Pelotas (Process 1.363.979). The informed consent was signed by all participants or guardians. Individuals identified as probable MDE (≥ 13 points in the EPDS) were encouraged to seek a health service and were given a list with possible locations for free psychological and psychiatric care in the city.

## RESULTS

In the 24 rural census tracts visited, 1,697 eligible individuals were identified, of which 1,453 answered the complete questionnaire on depression, resulting in 9% of losses and 5.4% of refusals. The proportion of non-responders was higher among men (p *<* 0.001) and among those aged 60 years or over (p *=* 0.017).

Table 1 describes the sample studied. Regarding the demographic characteristics, approximately half of the individuals were female; most of the sample was aged 60 years or over, self-declared as white, were married or living with a partner, and had between 5–8 years of study. Regarding work, most individuals worked in the rural area but did not develop rural activities. Regarding the time living in the rural area, more than half of the interviewees had always lived in rural areas. The average number of residents per household was 3.44 (information not shown in the Table) and, regarding the economic situation, more than half of the sample belonged to the socioeconomic class C. Regarding the behavioral and health variables, we highlight that: 16.8% of the interviewees were smokers, 8.7% reported at-risk drinking, 84.6% were insufficiently active in their leisure time, and 37.4% received a medical diagnosis of at least one morbidity ( Table 1 ).

**Table 1:** Description of the sample and prevalence of depressive symptomatology according to demographic, socioeconomic, behavioral, and health variables in rural residents. Pelotas, State of Rio Grande do Sul, Brazil, 2016. (n = 1,453)

Variable	n (%)	Prevalence of depressive symptomatology (95%CI)
Sex		
Male	701 (48.2)	25.2 (20.5–30.5)
Female	752 (51.8)	44.8 (40.6–49.0)
Age (years)		
18–29	280 (19.3)	33.6 (28.3–39.6)
30–39	225 (15.6)	35.7 (28.6–43.6)
40–49	292 (20.2)	37.6 (31.9–43.7)
50–59	286 (19.7)	38.6 (33.4–44.1)
≥ 60	370 (25.2)	32.0 (25.5–35.4)
Race		
White	1,241 (85.2)	34.1 (30.0–38.5)
Black, brown, or other	212 (14.8)	42.6 (35.8–49.8)
Marital status		
Married or living with a partner	889 (60.9)	34.2 (28.1–39.5)
Divorced, separated, widower	187 (12.9)	44.7 (38.0–51.7)
Single	377 (26.2)	33.5 (27.3–40.3)
Education level (complete years)		
0–4	536 (37.1)	40.0 (35.6–46.7)
5–8	546 (37.7)	35.6 (31.5–40.0)
≥ 9	364 (25.2)	27.6 (23.1–32.7)
Workplace ^a^		
Urban area	124 (14.1)	28.7 (21.9–38.9)
Rural area	703 (79.3)	33.9 (28.3–40.0)
Rural and urban area	60 (6.6)	34.6 (23.5–47.8)
Rural worker		
No	953 (65.9)	35.0 (31.2–39.1)
Yes	498 (34.1)	35.9 (29.2–43.2)
Time living in the rural area		
Short time (< 50% of life)	275 (19.2)	37.2 (30.6–44.3)
Long time (50%–99% of life)	203 (14.0)	33.8 (25.9–42.7)
Always (100% of life)	975 (66.8)	35.1 (31.2–39.3)
Number of residents in the household		
1	89 (6.1)	40.0 (29.3–51.6)
2	363 (25.1)	32.7 (26.9–39.1)
3	381 (26.6)	38.3 (32.2–44.7)
4	298 (20.3)	30.6 (24.0–38.0)
≥ 5	315 (21.9)	37.8 (33.1–42.8)
Socioeconomic class (ABEP)		
A or B	292 (20.2)	26.9 (23.0–31.3)
C	784 (54.1)	35.7 (31.1–40.5)
D or E	364 (25.7)	41.1 (34.4–39.3)
Smoking		
No	1,213 (83.2)	35.1 (30.9–39.6)
Yes	240 (16.8)	36.5 (29.0–44.7)
Pattern of alcohol consumption		
Low-risk drinking	1,328 (91.3)	35.7 (32.1–39.4)
At-risk drinking	125 (8.7)	32.0 (20.9–45.6)
Physical leisure activity		
Active (≥ 150 min/week)	221 (15.4)	28.3 (22.8–34.6)
Insufficiently active (< 150 min/week)	1,218 (84.6)	36.9 (32.9–41.0)
Number of morbidities ^b^		
0	905 (62.6)	31.7 (28.4–35.1)
1	405 (27.7)	39.9 (33.3–46.9)
2	120 (8.4)	44.4 (34.1–55.3)
3	19 (1.3)	57.9 (40.0–73.9)

ABEP: *Associação Brasileira de Empresas de Pesquisa* (Brazilian Association of Research Companies).

^a^ Variable with most missing data (n = 566).

^b^ Hypertension, diabetes, and heart disease.

The average EPDS score was 6.45 [standard deviation (SD) *=* 4.75, median *=* 6] (information not shown in the table). The prevalence of depressive symptomatology in the sample was 35.4% (95%CI 31.5–39.3). The crude and adjusted prevalence ratios of the outcome according to the independent variables are shown in Table 2 . In the crude analysis, the prevalence of depressive symptomatology was associated with: being female; white; divorced, separated, or widowed; having lower education level; belonging to socioeconomic classes D or E; being insufficiently active in the leisure time; and having a greater number of morbidities. After adjusting for confounding factors, the prevalence of depressive symptomatology in women was 1.77 times the prevalence in men (95%CI 1.46–2.15). The variables of education level and socioeconomic class remained associated after adjustment and presented an inverse relation with the outcome, that is, individuals with up to four years of education level and individuals with worse socioeconomic conditions (classes D or E) had the highest prevalence of depressive symptomatology compared to those with higher education level and better socioeconomic conditions (classes A or B).

**Table 2:** Crude and adjusted prevalence ratios for the association between depressive symptomatology (EPDS ≥ 8 points) and demographic, behavioral, and health variables in rural residents. Pelotas, State of Rio Grande do Sul, Brazil, 2016. (n = 1,453)

Variable	Crude PR	p ^c^	Adjusted PR	p ^c^
1st level				

Sex		< 0.001		< 0.001
Male	1.00		1.00	
Female	1.77 (1.48–2.13)		1.77 (1.46–2.15)	
Age (years)		0.454		0.172
18–29	1.05 (0.80–1.37)		1.36 (0.97–1.91)	
30–39	1.11 (0.88–1.40)		1.34 (0.98–1.85)	
40–49	1.17 (0.94–1.46)		1.35 (1.02–1.78)	
50–59	1.20 (0.93–1.56)		1.31 (1.00–1.72)	
≥ 60	1.00		1.00	
Race		0.029		0.062
White	1.00		1.00	
Black, brown, or other	1.25 (1.02–1.52)		1.19 (0.99–1.43)	
Marital status		0.001		0.337
Married/Living with a partner	1.00		1.00	
Divorced/Separated/Widowed	1.30 (1.13–1.50)		1.21 (0.92–1.57)	
Single	0.98 (0.75–1.26)		1.08 (0.85–1.36)	
Education level (complete years)		0.007 ^d^		0.002 ^d^
0–4	1.44 (1.11–1.87)		1.62 (1.22–2.16)	
5–8	1.28 (1.06–1.56)		1.39 (1.16–1.68)	
≥ 9	1.00		1.00	

2nd level				

Socioeconomic class (ABEP)		< 0.001 ^d^		0.001 ^d^
A or B	1.00		1.00	
C	1.32 (1.11–1.58)		1.36 (1.14–1.62)	
D or E	1.52 (1.24–1.86)		1.49 (1.22–1.83)	
Rural worker		0.809		0.469
No	1.00		1.00	
Yes	1.02 (0.83–1.26)		1.07 (0.87–1.33)	
Time living in the rural area		0.684		0.634
Short time	1.05 (0.87–1.28)		1.10 (0.87–1.39)	
Long time	0.96 (0.74–1.23)		0.96 (0.73–1.25)	
Always	1.00		1.00	
Number of residents in the household		0.391		0.293
1	1.05 (0.75–1.48)		1.03 (0.68–1.55)	
2	0.86 (0.73–1.02)		0.84 (0.64–1.12)	
3	1.01 (0.80–1.26)		1.01 (0.78–1.30)	
4	0.80 (0.63–1.03)		0.79 (0.60–1.05)	
≥ 5	1.00		1.00	

3rd level				

Smoking		0.747		0.744
No	1.00		1.00	
Yes	1.03 (0.81–1.33)		1.03 (0.82–1.31)	
Pattern of alcohol consumption		0.540		0.410
Low-risk drinking	1.00		1.00	
At-risk drinking	0.89 (0.62–1.28)		1.15 (0.80–1.66)	
Physical leisure activity ^a^		0.007		0.271
Active	1.00		1.00	
Insufficiently active	1.30 (1.08–1.56)		1.10 (0.92–1.32)	
Number of morbidities ^b^		< 0.001 ^d^		0.001 ^d^
0	1.00		1.00	
1	1.25 (1.08–1.46)		1.25 (1.05–1.50)	
2	1.40 (1.11–1.76)		1.41 (1.11–1.79)	
3	1.82 (1.30–2.55)		1.74 (1.24–2.45)	

ABEP: Associação Brasileira de Empresas de Pesquisa ( *Brazilian Association of Research Companies* ); EPDS: Edinburgh Postnatal Depression Scale

^a^ Variable with most missing data (n = 14).

^b^ Hypertension, diabetes, and heart disease.

^c^ Likelihood ratio test.

^d^ P-value of the linear trend test.

We also observed that the higher the number of chronic diseases, the greater the prevalence of depressive symptomatology. Subjects with hypertension, diabetes, and heart disease had a prevalence almost twice as high as those who did not report any of these diseases (OR *=* 1.74, 95%CI 1.24–2.45).

Although the variable of age was not statistically significant, the confidence intervals of the age groups from 40 to 59 years were significant and the measures of effect suggested higher prevalences in these age categories compared to the age group of 60 years or over.

Race, marital status, and leisure physical activity, although associated with depressive symptomatology in the crude analysis, lost statistical significance and the magnitude of the effect was reduced after adjusting for confounding factors. However, the variables of rural worker, time living in the rural area, and number of residents in the household and the behavioral variables (smoking and pattern of alcohol consumption) did not show statistical significance in the crude and adjusted analyses ( Table 2 ).

Additional analyses, considering the cutoff point of 13 or more points in the EPDS, show that the results found remained the same despite the more specific cutoff point to detect MDE, except for age and race, which remained associated after adjusting for confounding factors. The youngest individuals, aged between 18 and 29 years (PR *=* 2.11, 95%CI 1.12–3.98) and those self-declares as black, brown, or other (OR *=* 1.73, 95%CI 1.28–2.34) showed the highest prevalences for probable MDE ( Table 3 ).

**Table 3:** Crude and adjusted prevalence ratios for the association between probable MDE (EPDS ≥ 13 points) and demographic, behavioral, and health variables in rural residents. Pelotas, State of Rio Grande do Sul, Brazil, 2016. (n = 1,453)

Variable	Crude PR	p ^c^	Adjusted PR	p ^c^
1st level				

Sex		< 0.001		< 0.001
Male	1.00		1.00	
Female	2.92 (2.07–4.12)		2.79 (1.96–3.97)	
Age (years)		0.854		0.021 ^d^
18–29	1.02 (0.58–1.79)		2.11 (1.12–3.98)	
30–39	1.27 (0.76–2.11)		2.02 (1.16–3.51)	
40–49	1.16 (0.62–2.16)		1.62 (0.87–3.02)	
50–59	1.15 (0.72–1.85)		1.45 (0.94–2.23)	
≥ 60	1.00		1.00	
Race		< 0.001		0.001
White	1.00		1.00	
Black, brown, or other	1.87 (1.44–2.42)		1.73 (1.28–2.34)	
Marital status		< 0.001		0.118
Married/Living with a partner	1.00		1.00	
Divorced/Separated/Widowed	1.78 (1.35–2.36)		1.54 (0.99–2.39)	
Single	0.82 (0.54–1.25)		0.88 (0.56–1.37)	
Education level (complete years)		0.017 ^d^		0.005 ^d^
0–4	2.29 (1.06–4.97)		2.86 (1.28–6.39)	
5–8	1.72 (0.89–3.32)		1.93 (0.95–3.92)	
≥ 9	1.00		1.00	

2nd level				

Socioeconomic class (ABEP)		0.001 ^d^		0.005 ^d^
A or B	1.00		1.00	
C	1.77 (1.03–3.05)		1.92 (1.13–3.27)	
D or E	2.56 (1.46–4.51)		2.42 (1.37–4.27)	
Rural worker		0.809		0.612
No	1.00		1.00	
Yes	0.92 (0.67–1.27)		1.08 (0.77–1.54)	
Time living in the rural area		0.521		0.397
Short time (< 50% of life)	1.10 (0.65–1.85)		1.18 (0.76–1.81)	
Long time (50%–99% of life)	1.33 (0.80–2.19)		1.33 (0.86–2.06)	
Always (100% of life)	1.00		1.00	
Number of residents in the household		0.434		0.554
1	0.98 (0.50–1.89)		0.68 (0.31–1.46)	
2	0.75 (0.50–1.13)		0.70 (0.42–1.14)	
3	0.84 (0.54–1.30)		0.80 (0.42–1.14)	
4	0.73 (0.51–1.03)		0.68 (0.41–1.12)	
≥ 5	1.00		1.00	

3rd level				

Smoking		0.805		0.813
No	1.00		1.00	
Yes	1.05 (0.67–1.64)		1.04 (0.69–1.57)	
Pattern of alcohol consumption		0.399		0.550
Low-risk drinking	1.00		1.00	
At-risk drinking	0.76 (0.39–1.46)		1.22 (0.61–2.43)	
Physical leisure activity ^a^		0.114		0.440
Active	1.00		1.00	
Insufficiently active	1.72 (0.86–3.44)		1.26 (0.68–2.34)	
Number of morbidities ^b^		< 0.001 ^d^		0.007 ^d^
0	1.00		1.00	
1	1.49 (1.19–1.86)		1.44 (1.17–1.78)	
2	1.79 (1.25–2.56)		1.73 (1.13–2.64)	
3	2.26 (0.81–6.28)		1.66 (0.58–4.75)	

ABEP: Associação Brasileira de Empresas de Pesquisa ( *Brazilian Association of Research Companies* ); EPDS: Edinburgh Postnatal Depression Scale; MDE: major depressive episode

^a^ Variable with most missing data (n = 14).

^b^ Hypertension, diabetes, and heart disease.

^c^ Likelihood ratio test.

^d^ P-value of the linear trend test.

## DISCUSSION

More than a third of the individuals living in the rural area of Pelotas presented depressive symptomatology. International studies conducted in rural areas, in different contexts and with predominantly adult populations, have shown large variations in the prevalence of depressive symptomatology (between 5.1% and 56.2%) ^16–18^ . Therefore, comparisons between studies, although important, should consider that prevalences are different because of the socioeconomic, political, and cultural characteristics of the regions and the use of different instruments to evaluate depressive symptomatology.

In this study, the depressive symptomatology present in 35.4% of the individuals evaluated in the rural area was superior to those found in two studies carried out with the population living in the urban area of the same city ^12^ ^,^ ^19^ . Matijasevich et al. ^12^ have evaluated adults aged 20 years or over using EPDS, using the same cutoff point (≥ 8 points), and they have found a prevalence of 19% for depressive symptomatology. Another local study, using the Patient Health Questionnaire-9 (PHQ-9), observed a prevalence of 20.4% for this morbidity ^19^ . This comparison with the urban area of the city reinforces the importance of evaluating depressive symptomatology and the need for knowledge about the presence of this morbidity in populations living in rural areas.

As in other studies performed with rural residents ^18^ ^,^ ^20^ , we also found in this study a higher prevalence of depressive symptomatology in women. In the world, lifetime depression is most often found among women ^21^ ^,^ ^22^ . In general, men have a lower prevalence of depressive symptomatology and they can externalize these symptoms differently compared to women (with irritability and aggressiveness, for example) ^23^ . The reasons for the greater occurrence among women are still not completely clear; however, the existence of a combination of biological (such as hormonal) and environmental factors (inequalities, gender roles, domestic and civil violence) may explain some of these findings ^24^ . It is believed that women perform domestic activities to a greater extent than menin rural areas, invariably feeling more alone and pressured, less supported and recognized, and therefore more susceptible to developing depressive symptoms.

Regarding age group, studies with adults from rural regions of Australia and Lithuania have also shown higher prevalences of depressive symptomatology at intermediate ages ^17^ ^,^ ^18^ . In the rural area evaluated, the rate of non-respondents was higher among older adults, which may have underestimated the prevalence in this age group. In addition, it is believed that continuity at work, adaptation to the rural area, and therefore stability in the area may be important factors in explaining the lower prevalence of depressive symptomatology found among individuals aged 60 years or over when compared to younger persons.

Regarding education level and socioeconomic class, the depressive symptomatology in the rural area of Pelotas was higher among the less favored individuals, a frequent finding in the literature ^18^ ^,^ ^21^ . Individuals with lower education levels generally have a reduced range of choices and possibilities for social and economic advancement. These factors may lead to aggravation or development of mental disorders, such as depression ^24^ .

We also found that the prevalence of depressive symptomatology was higher among those with some evaluated chronic disease. This finding can be understood by the impact of this type of morbidity on the emotional life of the individual, which damages the social life and functional capacity ^25^ .

We found no association between smoking and depressive symptomatology in the rural population investigated. Studies show that smokers are more likely to develop depression and that those with a prior history of depression and anxiety tend to find it hard to quit smoking ^26^ ^,^ ^27^ . Furthermore, the use of tobacco causes physical and psychological dependence, pleasure, and relief of negative tensions and feelings ^28^ . The relation between smoking and depression should be better evaluated in future studies in the rural population; however, the possibility of reverse causality has to be considered.

We found the highest prevalence of depressive symptomatology among individuals who reported being black, brown, yellow, or indigenous. These individuals are, in general, a minority in the studied rural area, where most inhabitants are descendants of Germans and Pomeranians. However, this variable was only associated in the crude analysis.

In the adjusted analysis, we found no association between the presence of depressive symptomatology and occupation, or between depressive symptomatology and level of leisure physical activity. The lack of association may be related to the power of the study, which is insufficient to discriminate these differences, since the high amount of work performed by rural residents and the insufficient practice of leisure physical activity could contribute to the development of mental disorders. We also found no statistically significant difference for the variables: marital status, alcohol consumption, time living in the rural area, and number of residents in the household. More studies are needed to better explore these associations in the rural context.

Among the limitations of this study, we must consider the possibility of reverse causality, which is inherent in cross-sectional studies and hinders the establishment of a temporality relation between the outcome and some variables, previously scored, such as smoking. In addition, we did not question the use of antidepressants in the sample, which may have underestimated the prevalence found. We also highlight that the rate of non-respondents was higher among men and older adults, which may have reduced the prevalence of the outcome in these groups.

The instrument used to evaluate the outcome has been validated for the urban population ^12^ and we found no specific instrument to evaluate depression in residents of rural areas in the literature. However, the EPDS has been validated for the general population, of both sexes, living in the same city where this study was performed; therefore, because they live in the same region, the population of both places in the city (urban and rural) have a good understanding of the instrument, in addition to being similar in other ways. It is important to emphasize that, although the EPDS is useful to screen the disease, it does not replace the diagnosis based on a clinical interview performed by mental health professionals.

Among the positive aspects, we highlight that this is a population-based study with a low proportion of losses and refusals, with great methodological care during all the stages of the work ^10^ , and with a rigorous selection of individuals for a representative sample of the rural population. In this way, the results of this research can be used as reference for other rural regions with similar socioeconomic and cultural contexts.

This study highlights the importance of mental illness as a public health problem in rural areas. The creation of care programs aimed at rural residents for the early detection and diagnosis of depression and maintenance of treatment are important actions that should be fostered by local and national health policies.
